# Supplementary material for: Empirical methods for controlling false positives and estimating confidence in ChIP-Seq peaks
Source: BMC Bioinformatics. 2008 Dec 5;9:523. doi: 10.1186/1471-2105-9-523 (PMC2628906; doi:10.1186/1471-2105-9-523)
Supplement: Additional File 1 — A variety of html documents from the USeq web site detailing the available applications, their best usage, output file type descriptions, command line menus, etc. [file 1471-2105-9-523-S1.zip › USeqUserGuides/JavaDoc.zip!/JavaDoc/edu/utah/seq/data/class-use/SmoothingWindowInfo.html]

Uses of Class edu.utah.seq.data.SmoothingWindowInfo


|  |  |  |
| --- | --- | --- |
| **Overview** \| **Package** \| **Class** \| **Use** \| **Tree** \| **Deprecated** \| **Index** \| **Help** | |  |
| PREV   NEXT | **FRAMES**    **NO FRAMES**     **All Classes** |  |


---


## **Uses of Class edu.utah.seq.data.SmoothingWindowInfo**

| Packages that use SmoothingWindowInfo | |
| --- | --- |
| **edu.utah.seq.analysis** | Primary analysis applications and helper classes for working with binary PointData, xxx.bar.zip files. |
| **edu.utah.seq.data** | Helper classes for working with binary PointData, EnrichedRegions, etc. |

Uses of SmoothingWindowInfo in edu.utah.seq.analysis

Constructors in edu.utah.seq.analysis with parameters of type SmoothingWindowInfo

`EnrichedRegionMaker(int maxGap,
int scoreIndex,
SmoothingWindowInfo[] windowInfo)`

Uses of SmoothingWindowInfo in edu.utah.seq.data

| Methods in edu.utah.seq.data with parameters of type SmoothingWindowInfo | |
| --- | --- |
| `static float[]` | `SmoothingWindowInfo.minMax(SmoothingWindowInfo[] swi, int scoreIndex)`             Returns min and max of the scores for a given scoreIndex. |

---


|  |  |  |
| --- | --- | --- |
| **Overview** \| **Package** \| **Class** \| **Use** \| **Tree** \| **Deprecated** \| **Index** \| **Help** | |  |
| PREV   NEXT | **FRAMES**    **NO FRAMES**     **All Classes** |  |


---
